# Phenotypic characterization of nanshi oral liquid alters metabolic signatures during disease prevention

**DOI:** 10.1038/srep19333

**Published:** 2016-01-20

**Authors:** Aihua Zhang, Qi Liu, Hongwei Zhao, Xiaohang Zhou, Hui Sun, Yang Nan, Shiyu Zou, Chung Wah Ma, Xijun Wang

**Affiliations:** 1 National TCM Key Laboratory of Serum Pharmacochemistry, Laboratory of Metabolomics, Heilongjiang University of Chinese Medicine, Heping Road 24, Harbin 150040, China; 2 Research Center of Chinmedomics, Heilongjiang University of Chinese Medicine, Heping Road 24, Harbin 150040, China; 3 Infinitus (China) Company Ltd, Guangdong Province, China; 4 Department of Pharmaceutical Analysis, School of Pharmacy, Heilongjiang University of Chinese Medicine, Heping Road, Harbin, China

## Abstract

This paper was designed to investigate the phenotypic characterization of Nanshi Oral Liquid (NOL) alters metabolic signatures of the ‘Kidney Yang Deficiency syndrome’ (KYDS). Urine metabolites were profiled by UPLC-ESI-Q-TOF-HDMS. The significantly changed metabolites such as xanthurenic acid, 4,8-dihydroxyquinoline, 3-methyldioxyindole, 4,6-dihydroxyquinoline, kynurenic acid, hippuric acid, taurine, tyramine, and 3-metanephrine, had been identified, and were related to the disturbance in tyrosine metabolism, steroid hormone biosynthesis, taurine and hypotaurine metabolism, tryptophan metabolism, phenylalanine metabolism and lysine degradation, which were helpful to further understanding the KYDS and intervention mechanism of NOL. The biochemical result showed that NOL can alleviate the kidney impairment induced by KYDS. Metabolomics results indicated the significantly changed metabolites were found to be reasonable in explaining the action mechanism of NOL. Interestingly, the effectiveness of NOL against KYDS was proved using the established metabolomics method and regulated the biomarkers as well as adjusted the metabolic disorder pathways. NOL had potentially pharmacological effect through regulating multiple perturbed pathways to normal state. This work showed that the metabolomics method was a powerful approach for studying the phenotypic characterization of disease’s syndrome during disease prevention and its intervention mechanism.

Metabolomics, is an important method of systems biology, has recently been demonstrated significant potential in many fields such as drug discovery^1^, nutrition studies^2^ and toxicological investigation^3^. It determines the metabolites and then transforms the data of metabolic pattern into a useful information by pattern recognition tools, and assesses therapeutic effects of drug, and provides much valuable information on biochemical perturbations in mechanism-related information^4^. Metabolomics has been shown to have enormous potential in natural products discovery^5^,^6^. Currently, a number of analytical tools, including NMR spectroscopy^7^, UPLC/MS^8^,^9^,^10^, CE/MS^11^,^12^ and GC/MS^13^, have been used for metabolomics. The development of UPLC/MS technology and advanced analysis softwares^14^,^15^,^16^ lead to better chromatographic peak resolution, considerable shorter analysis time, high sensitivity and rapid identification, have been considered to have a more bright future in metabolomics. Recently, it has been increasingly used as a versatile tool for assessing therapeutic effects of many TCM prescriptions. TCM has a long history and been accepted by the academic community and patients as superior and a unique valuable property in China^17^. However, the advantages of TCM are difficult to understand because of the lack of the modern technologic approaches and the necessary molecular mechanism. Fortunately, metabolomics adopts a ‘top-down’ strategy to understand metabolic changes of a complete system caused by interventions in holistic context, suggesting that its property is in concert with the holistic efficacy of TCM^18^,^19^,^20^,^21^.

‘Kidney-yang deficiency syndrome’ (KYDS), one of the common syndrome patterns in TCM, recorded firstly in an earliest systematic and theoretical monograph existing in China, “Neijing”^22^, whose characteristics were warm dysfunction and metabolic disorder of body fluid, aversion to cold, chills, cold limbs, tinnitus, ache of waist and knee, impairment of hearing and looseness of teeth^23^,^24^. In the modern medicine research, KYDS was a complex kidney disorder involving many diseases such as nephrotic syndrome, chronic renal failure and the functional disorder with different degree of hypothalamic-pituitary-target gland (adrenal, thyroid and gonad) axis^25^,^26^. A classic method of the KYDS animal model was that the rat was injected with a high dose of corticosterone, made the hypothalamic-pituitary-target adrenal disordered, and which was used for investigating the mechanism and the preventative effect of KYDS^26^,^27^,^28^.

Nowadays, a new focus on the pursuit of TCM was sought for the treatment of KYDS. TCM, widely used for treatment of various diseases, recently had been attracted the interest of the world as alternative therapy. The most common clinical practice of TCM was herb combination called formula which consists of several types of medicinal herbs^29^. Nanshi Oral Liquid (NOL) was a Chinese herbal preparation, composed of *polygoni multiflori radix, corni fructus, orindae officinalis radix, rosae laevigatae fructus, jujubae fructus, juglandis semen* and *longan arillus*, which was used to treating KYDS diseases in China for decades. Nevertheless, it was impossible to explore the whole metabolites in KYDS and the action mechanism of NOL using the traditional ways. Metabolite profiling can provide the important evidences to elucidate the mechanism of herbal medicines. In light of this, we performed a UPLC/MS metabolomics approach to characterize the metabolic pathways of KYDS and to evaluate the intervention effect of NOL against KYDS.

## Results

### Biochemical analysis and histopathological observations

**Figure 1:**
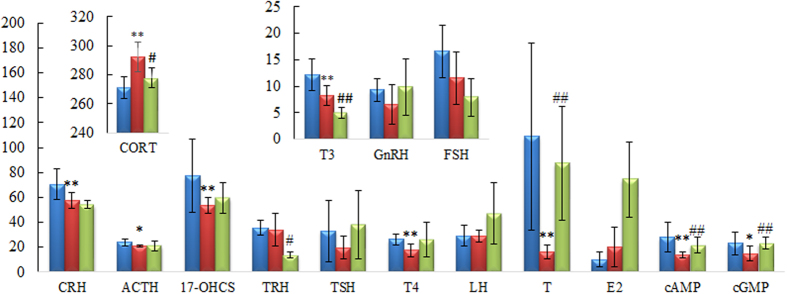
The biochemical characteristics. Bar plots represent the mean relative metabolite concentration and standard deviations. *significant difference from control at p < 0.05. **Significant difference from control at p < 0.01. ^#^Significant difference from model at p < 0.05. ^##^Significant difference from model at p < 0.01. The corresponding markers represented to the Supplementary Table 1.

**Figure 2:**
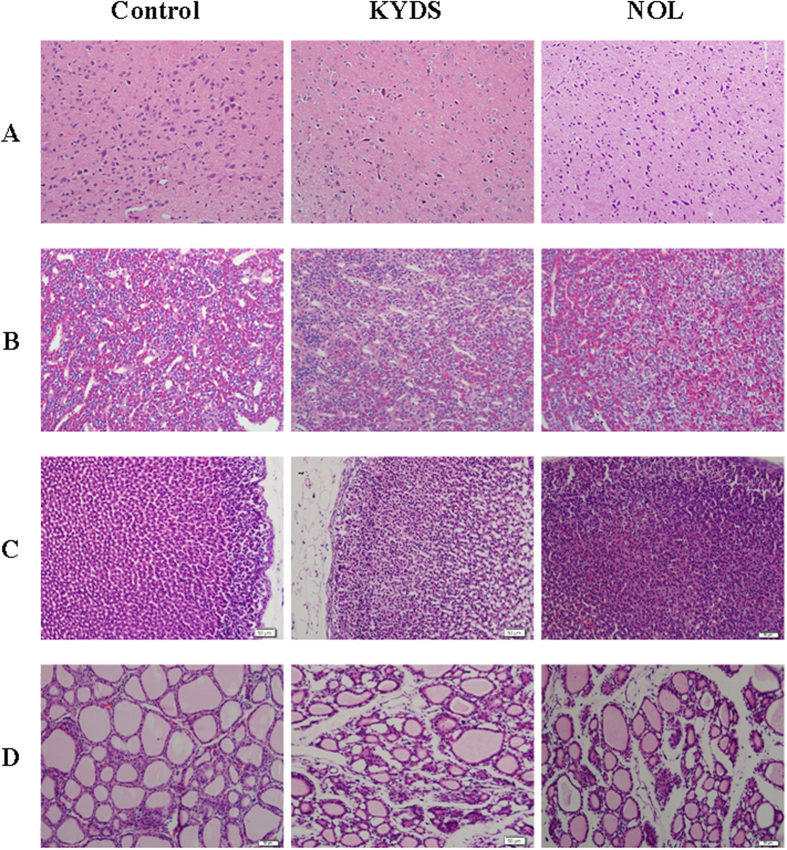
H&E staining for histological evaluation. Typical photographs of hypothalamic (**A**), pituitary (**B**), adrenal (**C**), thyroid (**D**) sections stained with H&E. (Magnification 200×).

The biochemistry parameters of the control group, KYDS group and NOL group were summarized in the supplementary Table 1 and Fig. 1. Compared with control group, a significant elevation of plasma corticosterone (CORT) and estradiol (E_2_) were observed in KYDS group, whereas the levels of corticotropin-releasing hormone (CRH), adrenocorticotropichormone (ACTH), 17-hydroxycorticosteroid (17-OHCS), thyroid-stimulating hormone (TSH), testosterone (T), cyclic adenosine monophosphate (cAMP) and cyclic guanosine monophosphate (cGMP) were decreased. The hormones of neuroendocrine immune system were decreased, which meant the neuroendocrine immune system was in a state of inhibition, all these results indicated the KYDS was successfully established. Meanwhile, disease severity was further verified by H&E staining of hypothalamic, pituitary, adrenal, thyroid sections (Fig. 2). Histopathological examination of control group, KYDS group and NOL group were clearly depicted. These morphological results showed that the number of the hypothalamic neurons was reduced and their morphology was atrophic; the ratio of basophilic cells to acidophilic cells was decreased; thyroid acinis were atrophic and transmutative, and the interstitial of them were fibroplasia in KYDS group, exhibited the typical pathological features of HPA and HPT inhibition. NOL group was prevented from the baseline levels of the KYDS group, which demonstrated that NOL had a prevention effect on the KYDS model. It can effectively ameliorate the KYDS symptoms as demonstrated by the marked in biochemical index.

### Multivariate statistical analysis of metabolite profiling

**Figure 3:**
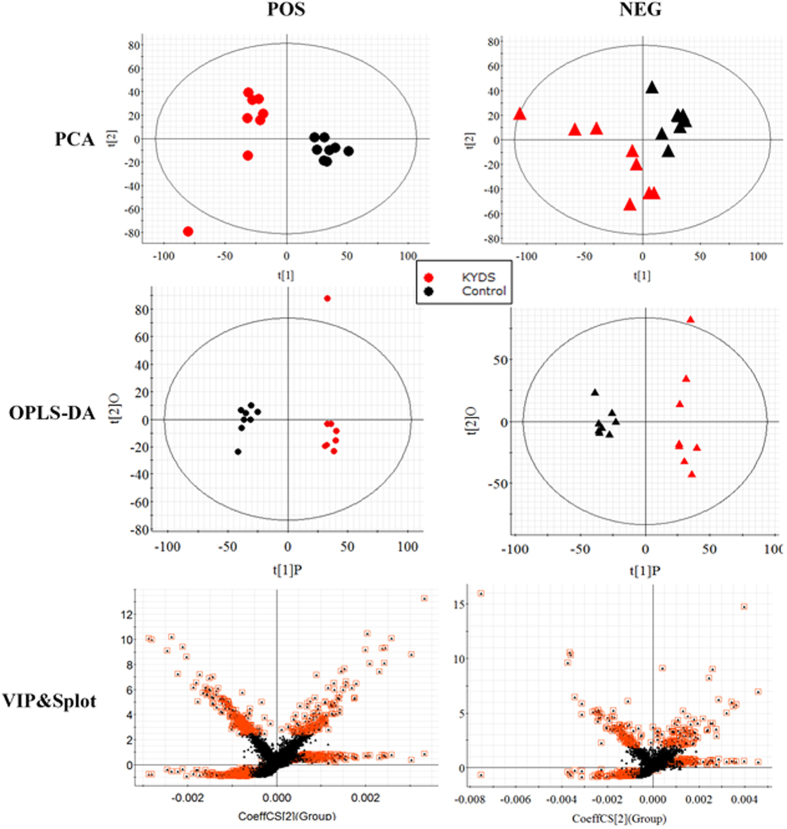
Multivariate data analyses resulting from the UPLC/MS spectra of urine samples. Score plots of PCA discriminating control group and KYDS group (**A**). OPLS-DA model for control group vs KYDS group in positive and negative ionisation mode (**B**). Potential biomarkers in the VIP&S-plot between control group and KYDS group (**C**)

Raw data from UPLC/MS were analysed by the Progenesis QI software. RT, m/z and peak height intensity were exported into EZinfo 2.0 software for data analysis. Multivariate data analysis was performed using the PCA method, and there was a distinguished classification between the clustering of the KYDS and control groups (Fig. 3). Obvious separation suggested that biochemical perturbation significantly happened in KYDS group. The VIP-plot and S-plot of OPLS were drawn to find biomarkers of KYDS in our study. The furthest metabolite ions from the origin exhibiting a higher value of VIP were potential biomarkers, and were responsible for the difference between control group and KYDS group. The ions that showed a significant difference in abundance between the control and KYDS animals were selected from the VIP-plot of OPLS-DA (Fig. 3).

### Metabolite identification

**Figure 4:**
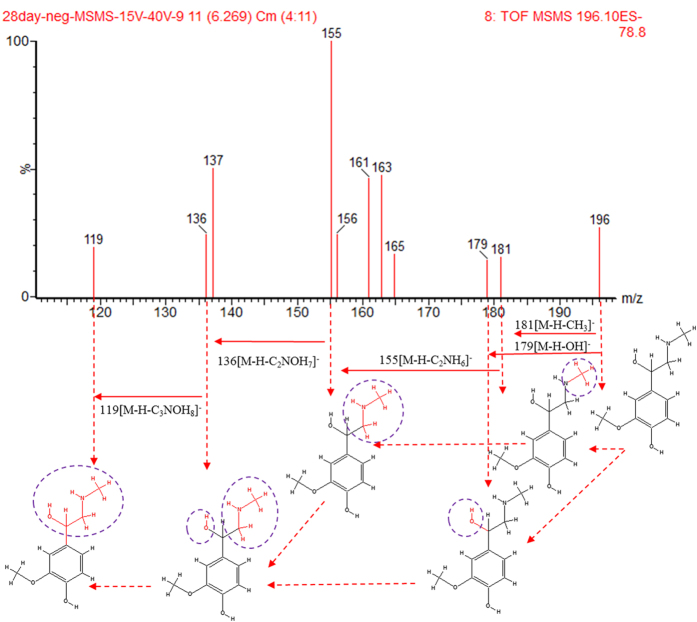
Chemical structure and mass fragment information of metanephrine in negative ionisation mode.

**Figure 5:**
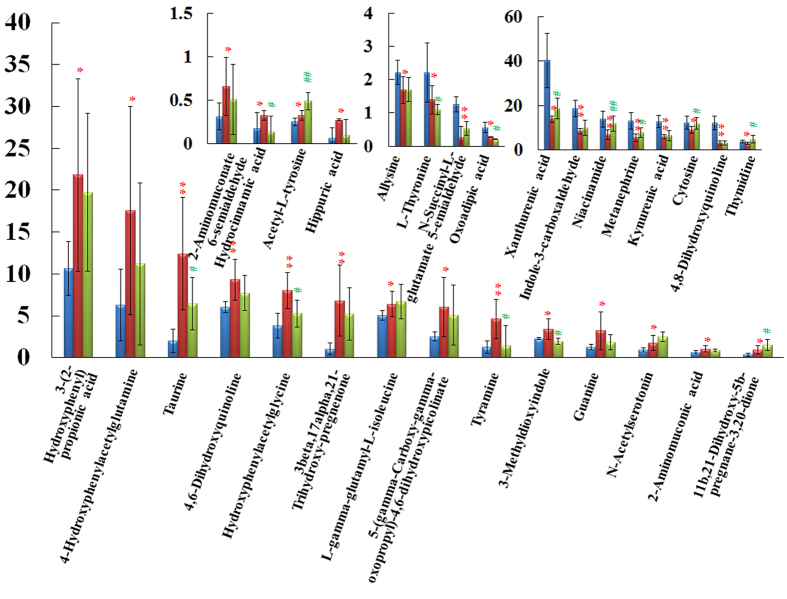
Relative signal intensities of the metabolites identified by UPLC/MS. The corresponding markers represented to the Supplementary Table 2. Bar plots represent the mean relative metabolite concentration and standard deviations. 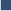: control group; 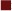: KYDS group; 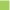:NOL group; *Significant difference from control at p < 0.05. **Significant difference from control at p < 0.01. ^#^Significant difference from model at p < 0.05.^##^Significant difference from model at p < 0.01.

**Figure 6:**
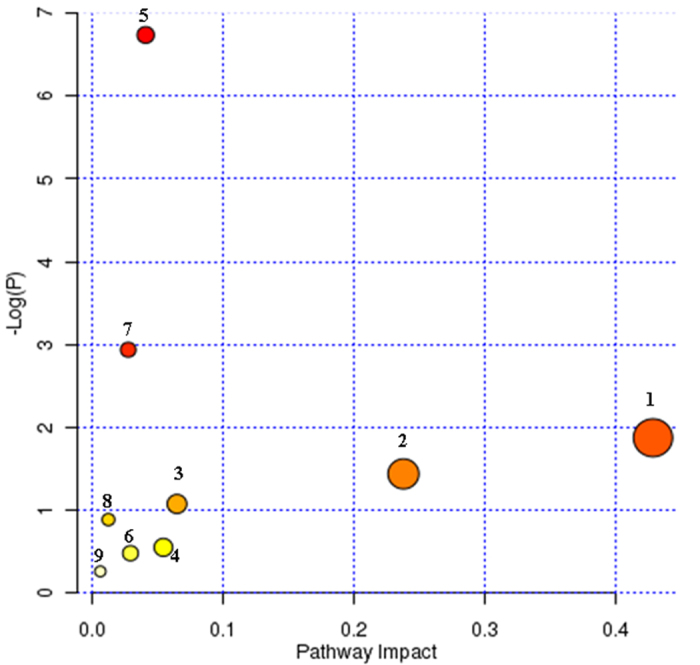
Summary of pathway analysis with MetPA tool. (1) tryptophan metabolism, (2) tyrosine metabolism, (3) taurine and hypotaurine metabolism, (4) nicotinate and nicotinamide metabolism, (5) lysine degradation, (6) purine metabolism, (7) pyrimidine metabolism, (8) primary bile acid biosynthesis, (9) steroid hormone biosynthesis.

Metabolite identification was conducted with high resolution MS, MS^E^ and MS/MS fragments, as well as database analyses. According the protocol described above, a number of potential biomarkers were identified and listed in supplementary material. In this study (Table S2), a total of 30 ions (VIP > 1.0, *p* < 0.05) contributed to the classification of the control group and KYDS group. These variables (18 in positive mode, 12 in negative mode) were predicted by comparing the accurate MS and MS^E^ with the metabolites found when searching Progenesis QI databases. Taking one ion as examples, the identification procedure was as follows. In the negative mode, the ion at Rt = 6.17 and [M−H]^−^ = 196.0965 has a high VIP value. This ion’s molecular formula was speculated as C_10_H_15_NO_3_ from the analysis of its elemental composition and fractional isotope abundance. The degree of unsaturation was calculated as 4, indicating that it was a ring compound. The main fragment ions analyzed by MS/MS screening were m/z 181, 179, 155, 136 and 119, which could be the [M−H]^−^ of lost –CH_3_, –OH, –C_2_NH_6_, –C_2_NOH_7_ and –C_3_NOH respectively. Finally, it was speculated as metanephrine when searching on-line databases, and its mass spectrum and structure were displayed in Fig. 4. 18 of 30 biomarkers were up-regulated and 12 of them were down-regulated in the KYDS group (Fig. 5). The related pathway of biomarker was identified by searching the KEGG database and the detailed construction of the metabolism pathways with higher score was shown in Table S3 and Fig. 6. Results suggested that (1) tryptophan metabolism, (2) tyrosine metabolism, (3) taurine and hypotaurine metabolism, (4) nicotinate and nicotinamide metabolism, (5) lysine degradation, (6) purine metabolism, (7) pyrimidine metabolism, (8) primary bile acid biosynthesis, (9) steroid hormone biosynthesis were involved in the pathological process of KYDS.

### Protective effects of NOL in KYDS syndrome

**Figure 7:**
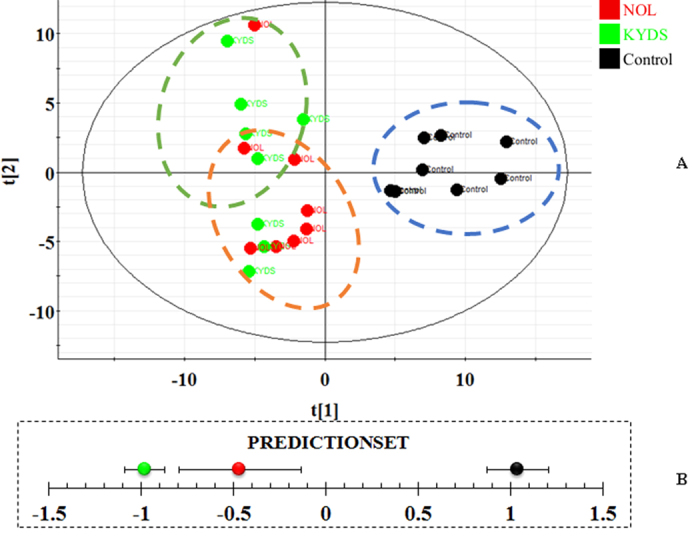
Score plots of PCA discriminating control group, KYDS group and NOL group (A). Result of intervention effect from predictionset modules (**B**).

**Figure 8:**
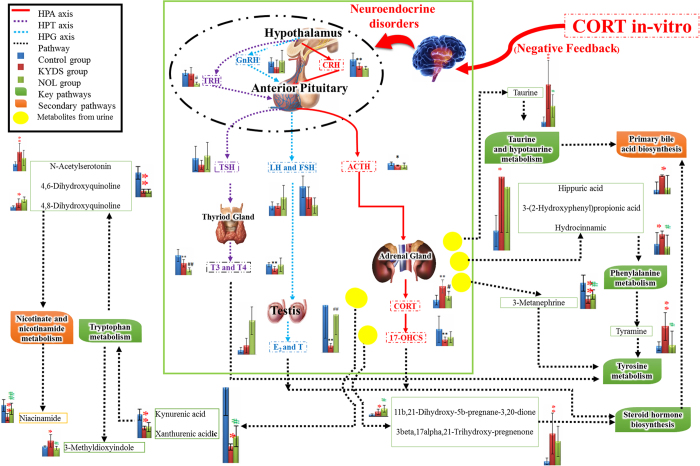
An overview of the perturbed metabolic pathways in response to KYDS and NOL treatment according to the KEGG. Th*ese* images were drawn by authors AZ and QL.

From the score plot of the PCA model, a clear separation among the control group, KYDS group and NOL group was easily seen, which was represented by an ellipse in Fig. 7, and NOL group was closer to the control group than the KYDS group, which suggested that NOL could reverse the pathological process of KYDS. By comparing the level of identified biomarkers in the control group, KYDS group and NOL group, 22 of them were completely reversed by NOL (Fig. 4), and the other metabolites were also reversed at different degrees. All of the reversed biomarkers belonged to the identified metabolism pathways. By relating the metabolic pathways, the metabolic network of the potential biomarkers was established (Fig. 8). Of note, NOL displayed an obvious anti-KYDS effect through adjusting the disturbed metabolism pathways such as tryptophan metabolism, taurine and hypotaurine metabolism, nicotinate and nicotinamide metabolism, tyrosine metabolism and phenylalanine metabolism, *etc*.

## Discussion

In order to clarify the mechanism of KYDS and the protective eeffects of NOL, we performed a UPLC/MS metabolomics approach to characterize the metabolic feature of KYDS and to evaluate the intervention effect of NOL against KYDS. Based on the support of the literatures^30^,^31^ we established KYDS rat. To validate the animal model of KYDS, a series of biochemistry parameters were given in Table S1. The concentration of CRH, 17-OHCS, T3, T4, T, cAMP, cGMP and ACTH were significantly decreased in KYDS rats, however, the CORT concentration was significantly increased (P < 0.01). From the Fig. 2, we found that the neurons of hypothalamic, zona fasciculation cells of the adrenal glands, thyroid follicular and the epithelial cell of thyroid follicular were atrophic in KYDS rats. Furthermore, the state of the KYDS rats were observed, and we found that they gradually lost weight, became weak, inactive, dehairing, and curled. All these results indicated that the rats presented the typical pathological features of KYDS. The biological parameters suggested that intervention effect of NOL on the regulation of the hypothalamic-pituitary-target gland axis was multiple potential sites.

Next, we had explored novel marker metabolites to understanding KYDS and protective mechanism of NOL using metabolomics approach. The PCA result was showed in Fig. 7, and a clear separation of KYDS and control group was achieved. The levels of 30 metabolites exhibited the marked changes compared with that of control group, which were related to taurine and hypotaurine metabolism, nicotinate and nicotinamide metabolism, lysine degradation, pyrimidine metabolism, tryptophan metabolism, primary bile acid biosynthesis, tyrosine metabolism, purine metabolism and steroid hormone biosynthesis. After orally administered with NOL for the following 21 days, it could prevent the changes of 17-OHCS, T4, T, cAMP, cGMP and ACTH (Fig. 1). Metabolomics analysis results showed that NOL group could prevent the levels of marker metabolites toward that of KYDS group. Figure 7 was displayed the relative intensities of the metabolites in three groups, which showed that after the administration of NOL, the levels of marker metabolites tended to that of the control group, and demonstrated that NOL could prevent KYDS development. After treatment with NOL, the relative content of these metabolites were effectively regulated, which suggested that the prevention effect of NOL against KYDS might involve regulating the phenylalanine metabolism, tyrosine metabolism, steroid hormone biosynthesis, taurine and hypotaurine metabolism.

In this study, KYDS was characterized by increasing in the 3-methyldioxyindole, 4,6-dihydroxyquinoline, 5-(gamma-carboxy-gamma-oxopropyl)-4,6-dihydroxypicolinate, N-acetyl-serotonin, and decreasing in the xanthurenic acid, 4,8-dihydroxyquinoline, indole-3-carboxaldehyde and kynurenic acid, which were related to tryptophan metabolism. Kynurenic acid and xanthurenic acid were noteworthy due to their metabolic processes^32^,^33^. It was reported that kynurenic acid was down-regulated in patients with chronic kidney disease^34^,^35^. Meanwhile, the other metabolites, such as 4,6-dihydroxyquinoline and N-acetylserotonin were the metabolite of 5-hydroxytryptophane involving the tryptophan metabolism, reflected a level of central nervous activity. N-acetylserotonin was a metabolic intermediate in melatonin synthesis, and several studies^36^,^37^ showed that it might have cytoprotective effects due to its antioxidant properties. The increase of N-acetylserotonin in KYDS rats showed that nervous system disorders. All these metabolite metabolism speculated that the pathological mechanism of KYDS was related to the kidney disease and nervous system disorders.

Taurine, an important marker metabolites, played a key role in the regulation of intracellular free calcium concentration^38^. On the other hand, taurine and γ-aminobutyric acid were two amino acids which were similar in chemical structure and both had been presented as inhibitory neurotransmitters or modulators in the mammalian central nervous system^39^. In a study, Teng Mu *et al.* had proved that the taurine could improve the function of HPG axis^40^. Meanwhile, excessive excretion of taurine into the urine was found in several clinical situations, including renal tubular damage and renal immaturity^41^. In our research, we found the increasing level of taurine in KYDS than that of control group, suggesting taurine had been dseverely influenced.

Tyramine, acetyl-l-tyrosine, 3-metanephrine and 4-hydroxyphenylacetylglutamine, the metabolites of tyrosine metabolism, contributed to the separation between the KYDS group and the control group. Tyramine is a key metabolite that connected tyrosine metabolism and phenylalanine metabolism, and synthesized from phenylalanine and converted into dopamine by tyrosine hydroxylase and aromatic amino acid decarboxylase in brain^42^. Then dopamine can be converted into catecholamine, such as norepinephrine (noradrenaline) and epinephrine (adrenaline). Epinephrine and norepinephrine also could activate the HPA axis by activating the CRH in the hypothalamus. Thus, this biochemical process connected the HPA axis hormone synthesis mechanism^43^,^44^. Meanwhile, the thyroid hormones T3 and T4 were derived from tyrosine^45^. From the result in Figs 1 and 5, we discovered the content of acetyl-L-tyrosine, the precursor of tyramine, and tyramine increased significantly, together with decreases in 3-metanephrine, 4-hydroxyphenylacetylglutamine, T3 and T4 were statistically significant in KYDS compared with that of control group. KYDS was disordered in HPA and HPT axis which had been confirmed by the results of clinical chemistry indexes, and also confirmed by the experimental work of metabolomics, from which we found the key pathways of KYDS was related to tyrosine metabolism and phenylalanine metabolism. This requires further indepth study. Combined metabolomics results with the pharmacological assay, we can obtain a better knowledge on the preventive effects of the NOL via regulating the disturbed metabolism pathways. Our work also confirmed the metabolomics platforms to dissecting the underlying efficacies and protective mechanisms of TCM.

Here, we have reported of metabolic patterns of KYDS by urinary metabolomics approach. 30 differential metabolites were identified and associated with KYDS. More importantly, 22 of them were completely reversed by NOL. Interestingly, tyrosine metabolism, steroid hormone biosynthesis, taurine and hypotaurine metabolism, tryptophan metabolism, and lysine degradation were the most altered functional pathways associated with KYDS according to ingenuity pathway analysis. In summary, this study suggested that the metabolomics approach was a useful tool which contributed further understanding of disease mechanisms, and provided helpful information for further pharmacological research of NOL.

## Experimental Work

### Materials and reagents

Acetonitrile, HPLC grade, was obtained from Merck (Darmstadt, Germany); methanol (HPLC grade) was purchased from Fisher Scientific Corporation (Loughborough, UK); ultrapure water was produced by a Milli-Q Ultra-pure water system (Millipore Corporation, MA, USA); leucine enkephalin was purchased from Sigma-Aldrich (St. Louis, MO, USA). NOL was provided by Infinitus (China) Company Ltd (Guangzhou, China). Corticosterone was provided by Sigma-Aldrich (St. Louis, MO, USA. Corticotropin releasing hormone (CRH), corticosterone (CORT), adrenocorticotropic hormone (ACTH) kit were provided by Beijing Huaying biological Ltd.; thyroid stimulating hormone (TSH), triiodothyronine (T3), thyroxine (T4), luteinizing hormone (LH), testosterone (T), estradiol (E_2_) kit were provided by Beijing North Biotechnology Institute; cAMP kit, cGMP kit, gonadotropin-releasing hormone (GnRH), thyrotropin-releasing hormone (TRH) kit were provided by R&D. All other reagents were of analytical grade.

### Animal handling

Male Wistar rats (weighting 250 ± 20 g) were provided by Weitong-Lihua Experimental Animal Center (Beijing, China). They had free access to food pellets and tap water under standard conditions of relative humidity (50 ± 5%), temperature (25 ± 1 °C) and 12 h light-dark cycle. All animals were allowed to acclimatize in metabolism cages for 1 week prior to treatment. After acclimatisation, all the rats were randomly divided into 3 groups of 8 rats each as follows: control group, model group, and NOL-treated group. They were treated as follows: from the 1^st^ day to 7^th^ day, NOL pretreatment group was orally administered NOL (dissolved in saline) at a dose of 1 mL/100 g once day, meanwhile, control group and KYDS group were orally administered saline at a dose of 1 mL/100 g once day; from the 8^th^ day to 28^th^ day, NOL group was orally administered NOL at a dose of 1 mL/100 g once day and injected subcutaneously corticosterone (10 mg/mL, dissolved in olive oil) at a dose of 1 mL/kg once day, meanwhile, control group and KYDS group were orally administered saline at a dose of 1 mL/100 g once day; however, KYDS group was injected subcutaneously corticosterone at a dose of 1 mL/kg once day, and control group was subcutaneously injected olive oil at a dose of 1 mL/kg once day. The experimental protocol was approved by the Animal Care and Use Committee of Heilongjiang University of Chinese Medicine (HUCM-2014-08717). The experimental methods were conducted according to the principles expressed in the Declaration of Helsinki.

### Collection and preparation of biosamples

On the 28^th^ day, urine was collected for 12h from metabolism cages at ambient temperature throughout the whole procedure and centrifuged at 13,000 rpm at 5 °C for 15 min, and the supernatant was stored frozen at −80 °C until metabolomics analysis. After collecting urine samples, all rats were deeply anesthetized and then blood was collected from the abdominal aorta and was immediately transferred into tubes and centrifuged at 3000 rpm for 10 min at 4 °C. Before all rats were sacrificed, the hypothalamic, pituitary, adrenal and thyroid sections were quickly taken out and fixed in 10% formalin. Supernatant samples were collected and stored at −80 °C flash frozen in liquid nitrogen until analyses; the serum was used for biochemical assay followed manufacturer’s instructions on commercial kits.

### Clinical chemistry and histopathology analysis

Biochemical parameters of plasma were analyzed on an automatic analyzer. The hypothalamic, pituitary, adrenal and thyroid sections were stained with hematoxylin and eosin (H&E), and reviewed by light microscopy. Image analysis was performed using Motic Medical 6.0 software (Xiamen Motic Software Engineering Co., Ltd). The histopathology analysis was done by the affiliated hospital of Heilongjiang University of Chinese Medicine.

## Metabolic Profiling

### Ultra-performance liquid chromatography

Chromatographic separation was performed on an ACQUITY UPLC system (Waters Corporation, Milford, MA) consisting of a binary solvent manger, a sample manager and a column compartment. The column used was an HSS C_18_ column (100 mm × 2.1 mm i.d., 1.8 μm, Waters Corporation, Milford, USA). Column temperature was maintained at 40 °C for all analyses. The optimal mobile phase consisted of a linear gradient system of (A) 0.1% formic acid in acetonitrile and (B) 0.1% formic acid in water: 0 to 1 min, 1% A; 1 to 2.5 min, 1 to 13% A; 2.5 to 6.5 min, 13 to 40% A; 6.5 to 8.0 min, 40 to 99% A; 8.0 to 10.5 min, 99% A; 10.5 to 11.0 min, 99 to 1% A; 11 to 13 min, 1 A. The flow rate was set to 0.4 mL/min. Injection volume was 3 μL. All the samples were kept at 4 °C during the analysis. In addition, the quality control sample was used to optimise the condition of UPLC, as it contained most information of whole urine samples.

### Mass spectrometry

High-definition mass spectrometry was performed on a Waters Q-TOF (SYNAPT™, Waters Corp, Manchester, England) equipped with an electrospray ion source in the positive and negative ionisation mode. The optimal conditions of analysis were as follow: the source temperature was set to 110 °C, desolvation gas temperature was 450 °C, cone gas flow was 50 h, desolvation gas flow was 600 L/h. In positive ion mode, the capillary voltage was 3.0 kV, the sampling cone voltage was 25 V, and extraction cone voltage was 3.0 V, desolvation gas flow was 600 L/h. In negative ion mode, the capillary voltage was 3.0 kV, the sampling cone voltage was 20 V, and extraction cone voltage was 4.0 V, desolvation gas flow was 700 L/h. Data of urinary samples were collected in the centroid mode between m/z 50 and 1000, with a scan time of 0.4 s and interscan time 0.1 s. For a part of QC samples were collected in the MS^E^ mode, performed on a Waters Q-Tof Premier mass spectrometer set at 15 eV for low collision energy and 35 eV for high collision energy, used to identify the compounds with Progenesis QI. For data accuracy and reproducibility, all analyses were carried out with an independent reference spray via the LockSpray interference. Leucine enkephalin at a concentration of 0.2 ng/mL was used via a lock spray interface at a flowrate of 100 μl·min^−1^ monitoring for positive ion mode ([M+H]^+^ = 556.2771) and negative ion mode ([M-H]^−^ = 554.2615) to ensure accuracy during the MS analysis. Lock spray frequency was set at 10 s and scan to average for correction was 10 s with the reference cone voltage at 35 V.

### Data processing and Multivariate data analyses

For quantitative metabolomics, raw data files and MS^E^ data files were uploaded onto Progenesis QI 1.0 software (Nonlinear Dynamics, 2014, version:1.0). Chromatographic alignment (with additional manual manipulation), data normalization (with normalize to all compounds) and peak picking (with retention time (RT) and mass to charge ratio (m/z) data pairs) were performed by Progenesis QI. A three-dimensional matrix was constructed and then exported into EZinfo 2.0 software for multivariate data analyses. Pareto scaling transformation was applied to the data processing before principal component analysis (PCA) and orthogonal partial least square discriminant analysis (OPLS-DA) were performed. Variables of interest were extracted from VIP-plots constructed with OPLS-DA, were considered as potential biomarkers. These potential biomarkers ions were transferred into Progenesis QI and marked with tag 1, meanwhile, added tag 2 to the ions in tag 1, when the test p value was less than 0.05, subjected to further identification of the molecular formula.

### Identification of biomarkers and metabolic pathway

The molecular ions that in tag 2 were found from MS^E^ data files with the MS/MS information in Progenesis QI. The compound identification list, contained the molecular weight, the name, the score, and other information to show the result of the identifications, was exported as an excel file. To check and confirm the identifications, the databases of HMDB, ChemSpider and KEGG were used by comparing molecular weights and MOL files. The molecular and structural formulas of the candidate compounds were retrieved by the comparison and then confirmed by MS/MS scans for the characteristic ions and fragmentation patterns of the metabolites. The construction, interaction and pathway analysis of potential biomarkers were performed with MetaboAnalyst software based on database source including the KEGG and HMDB.

### Statistical analysis

SPSS 17.0 for Windows was used for the statistical analysis of the biochemical data. The PCA was used to uncover unknown trends in the treated groups. Statistically significant differences in mean values were tested by using Student’s t-test, and p < 0.05 was considered statistically significant. Prior to multivariate analysis, the resultant data matrices from LC-MS data were mean-centered. Prediction set modules in the OPLS-DA mode were used to show the detail of intervention effect of each sample.

## Additional Information

**How to cite this article**: Zhang, A. *et al.* Phenotypic characterization of nanshi oral liquid alters metabolic signatures during disease prevention. *Sci. Rep.* **6**, 19333; doi: 10.1038/srep19333 (2016).

## Supplementary Material

Supplementary Information
