# Supplementary material for: Phenotypic characterization of nanshi oral liquid alters metabolic signatures during disease prevention
Source: Sci Rep. 2016 Jan 20;6:19333. doi: 10.1038/srep19333 (PMC4726315; doi:10.1038/srep19333)
Supplement: Supplementary Information [file srep19333-s1.doc]

**Phenotypic characterization of nanshi oral liquid alters metabolic signatures during disease prevention**

Aihua Zhang^1,2‡^ , Qi Liu^1,2‡^, Hongwei Zhao^3^, Xiaohang Zhou^1,2‡^, Hui Sun^1,4‡^, Yang Nan^1,2‡^, Shiyu Zou^3^, Chung Wah Ma^3^, Xijun Wang^1,2^*

^1^ National TCM Key Laboratory of Serum Pharmacochemistry, Laboratory of Metabolomics, Heilongjiang University of Chinese Medicine, Heping Road 24, Harbin 150040, China.

^2^ Research Center of Chinmedomics, Heilongjiang University of Chinese Medicine, Heping Road 24, Harbin 150040, China.

^3^ Infinitus (China) Company Ltd, Guangdong Province, China

^4^ Department of Pharmaceutical Analysis, School of Pharmacy, Heilongjiang University of Chinese Medicine, Heping Road, Harbin, China.

*Correspondence

Prof. PhD. Xijun Wang

National TCM Key Laboratory of Serum Pharmacochemistry

Key Laboratory of Metabolomics

Research Center of Chinmedomics

Heilongjiang University of Chinese Medicine

Heping Road 24, Harbin 150040, China

Tel. & Fax +86-451-82110818

Email: chinmedomics@126.com

^‡^ These authors contributed equally to this work.

**Table S1**. The biochemical characteristics results of KYDS after NOL treatment.

| Groups | CRH/  ng/dl | ACTH/  pg/ml | CORT/  ng/ml | 17-OHCS  /Umol/L | TRH/  Umol/L | TSH/  nIU/ml | T3/  ng/dl | T4/  ng/ml |
| --- | --- | --- | --- | --- | --- | --- | --- | --- |
| Control | 70.4±12.3 | 23.79±3.02 | 271.0±7.47 | 77.26±29.43 | 35.6±5.72 | 33.00±24.5 | 12.20±2.95 | 26.2±4.1 |
| KYDS | 57.4±6.1** | 20.89±0.71* | 292.3±10.0** | 53.45±6.6** | 33.9±13.07 | 19.69±9.4** | 8.27±1.89** | 17.39±5.09** |
| NOL | 54.2±3.3 | 21.07±4.02 | 277.9±6.68# | 59.24±12.4 | 13.55±2.29## | 38.2±27.2 | 4.92±1.05## | 26.29±13.8 |

**Table S1 (continued)**

| Groups | GnRH/  ng/ml | FSH/  μIU/dl | LH/  mIU/dl | T/  pg/ml | E_2_/  nmol/dl | cAMP/  ng/ml | cGMP/  pg/ml |
| --- | --- | --- | --- | --- | --- | --- | --- |
| Control | 9.35±2.16 | 16.60±4.89 | 29.2±8.5 | 108.96±75.19 | 10.13±5.97 | 27.78±11.89 | 23.06±8.93 |
| KYDS | 6.59±3.67 | 11.53±5.01 | 28.9±4.5 | 16.25±5.6** | 20.05±15.75 | 13.68±2.37** | 14.92±5.67* |
| NOL | 9.93±5.32 | 7.89±3.61 | 46.9±24.6 | 87.2±45.6## | 74.2±30.3 | 21.7± 6.13## | 23.27±4.64## |

Note:

* significant difference from control at p < 0.05.

** Significant difference from control at p < 0.001.

# Significant difference from model at p < 0.05.

## Significant difference from model at p < 0.001.

**Table S2. Identification of potential biomarkers of KYDS based on urine metabolic profiling.**

| **No.** | **Rt (min)** | **m/z determined** | **m/z calculated** | **Error (ppm)** | **Ion form** | **Formula** | **Metabolite Name** | **Trend** | **VIP Value** |
| --- | --- | --- | --- | --- | --- | --- | --- | --- | --- |
| 1 | 1 | 254.0313 | 254.0301 | 4.7 | [M-H]- | C_10_H_9_NO_7_ | 5-(gamma-Carboxy-gamma-oxopropyl)-4,6-dihydroxypicolinate | ↑ | 2.30879 |
| 2 | 1.27 | 138.0918 | 138.0919 | 0.7 | [M+H]+ | C_8_H_11_NO | Tyramine | ↑ | 3.0861 |
| 3 | 1.88 | 142.0508 | 142.0504 | 2.8 | [M+H]+ | C_6_H_7_NO_3_ | 2-Aminomuconate 6-semialdehyde | ↑ | 3.2266 |
| 4 | 2.74 | 152.0578 | 152.0572 | 3.9 | [M+H]+ | C_5_H_5_N_5_O | Guanine | ↑ | 1.8199 |
| 5 | 3 | 261.1453 | 261.145 | 1.1 | [M+H]+ | C_11_H_20_N_2_O_5_ | L-gamma-glutamyl-L-isoleucine | ↑ | 1.1476 |
| 6 | 3.23 | 162.0556 | 162.0555 | 0.6 | [M+H]+ | C_9_H_7_NO_2_ | 4,6-Dihydroxyquinoline | ↑ | 2.3549 |
| 7 | 3.3 | 208.0606 | 208.061 | -1.9 | [M-H]- | C_10_H_11_NO_4_ | Hydroxyphenylacetylglycine | ↑ | 2.8523 |
| 8 | 3.35 | 162.0558 | 162.0555 | 1.9 | [M-H]- | C_9_H_9_NO_2_ | 3-Methyldioxyindole | ↑ | 1.2445 |
| 9 | 3.58 | 151.0755 | 151.0759 | -2.6 | [M+H]+ | C_9_H_10_O_2_ | hydrocinnamic acid | ↑ | 2.0171 |
| 10 | 3.59 | 280.0846 | 280.0845 | 0.5 | [M-H]- | C_13_H_15_NO_6_ | 4-Hydroxyphenylacetylglutamine | ↑ | 3.9203 |
| 11 | 4.18 | 180.0698 | 180.0694 | 2.2 | [M+H]+ | C_9_H_9_NO_3_ | Hippuric acid | ↑ | 2.0385 |
| 12 | 4.21 | 224.0918 | 224.0923 | -2.2 | [M+H]+ | C_11_H_13_NO_4_ | Acetyl-L-tyrosine | ↑ | 1.6481 |
| 13 | 4.41 | 219.1137 | 219.1134 | 1.4 | [M+H]+ | C_12_H_14_N_2_O_2_ | N-Acetylserotonin | ↑ | 1.2422 |
| 14 | 4.44 | 126.0224 | 126.0225 | -0.8 | [M+H]+ | C_2_H_7_NO_3_S | Taurine | ↑ | 4.80923 |
| 15 | 5.11 | 165.0551 | 165.0552 | -0.6 | [M-H]- | C_9_H_10_O_3_ | 3-(2-Hydroxyphenyl)propionic acid | ↑ | 4.0185 |
| 16 | 5.28 | 349.239 | 349.2379 | 3.1 | [M+H]+ | C_21_H_32_O_4_ | 11b,21-Dihydroxy-5b-pregnane-3,20-dione | ↑ | 1.579 |
| 17 | 5.72 | 349.2374 | 349.2379 | -1.4 | [M+H]+ | C_21_H_32_O_4_ | 3beta,17alpha,21-Trihydroxy-pregnenone | ↑ | 2.8392 |
| 18 | 4.74 | 158.0442 | 158.0453 | -7 | [M+H]+ | C_6_H_7_NO_3_ | 2-Aminomuconic acid | ↑ | 1.2283 |
| 19 | 4.01 | 274.1093 | 274.1079 | 5.1 | [M+H]+ | C_15_H_15_NO_4_ | L-Thyronine | ↓ | 1.4345 |
| 20 | 0.8 | 243.0988 | 243.0981 | 2.9 | [M+H]+ | C_10_H_14_N_2_O_5_ | Thymidine | ↓ | 1.9671 |
| 21 | 1.01 | 123.0561 | 123.0558 | 2.4 | [M+H]+ | C_6_H_6_N_2_O | Niacinamide | ↓ | 4.5512 |
| 22 | 1.1 | 159.029 | 159.0293 | -1.9 | [M-H]- | C_6_H_8_O_5_ | Oxoadipic acid | ↓ | 1.0601 |
| 23 | 1.26 | 112.0513 | 112.0511 | 1.8 | [M+H]+ | C_4_H_5_N_3_O | Cytosine | ↓ | 2.8927 |
| 24 | 2.4 | 230.0664 | 230.0665 | -0.4 | [M-H]- | C_9_H_13_NO_6_ | N-Succinyl-L-glutamate 5-semialdehyde | ↓ | 1.13156 |
| 25 | 2.53 | 144.0668 | 144.0661 | 4.9 | [M-H]- | C_6_H_11_NO_3_ | Allysine | ↓ | 2.5583 |
| 26 | 3.46 | 206.0443 | 206.0453 | -4.9 | [M+H]+ | C_10_H_7_NO_4_ | Xanthurenic acid | ↓ | 7.2489 |
| 27 | 3.52 | 160.0396 | 160.0399 | -1.9 | [M-H]- | C_9_H_7_NO_2_ | 4,8-Dihydroxyquinoline | ↓ | 4.5192 |
| 28 | 3.75 | 144.0446 | 144.0449 | -2.1 | [M-H]- | C_9_H_7_NO | Indole-3-carboxaldehyde | ↓ | 10.836 |
| 29 | 3.75 | 188.0349 | 188.0348 | 0.5 | [M-H]- | C_10_H_7_NO_3_ | Kynurenic acid | ↓ | 5.9336 |
| 30 | 6.17 | 196.0965 | 196.0974 | -4.6 | [M-H]- | C_10_H_15_NO_3_ | Metanephrine | ↓ | 3.52 |

Note:“↑” and “↓” represent the compound is up- and down-regulated.

**Table S3**. Ingenuity pathway analysis with MetPA tool.

| **Pathway Name** | **Total Cmpd** | **Expected** | **Hits** | **Raw p** | **Holm p** | **FDR** | **Impact** |
| --- | --- | --- | --- | --- | --- | --- | --- |
| Taurine and hypotaurine metabolism | 8 | 0.1654 | 1 | 0.1543 | 1 | 1 | 0.4286 |
| Nicotinate and nicotinamide metabolism | 13 | 0.2689 | 1 | 0.2388 | 1 | 1 | 0.2381 |
| Lysine degradation | 20 | 0.4136 | 1 | 0.3436 | 1 | 1 | 0.0652 |
| Pyrimidine metabolism | 41 | 0.8481 | 1 | 0.5809 | 1 | 1 | 0.0549 |
| Tryptophan metabolism | 41 | 0.8481 | 5 | 0.0012 | 0.0956 | 0.0956 | 0.0414 |
| Primary bile acid biosynthesis | 46 | 0.9515 | 1 | 0.6237 | 1 | 1 | 0.0298 |
| Tyrosine metabolism | 42 | 0.8688 | 3 | 0.05319 | 1 | 1 | 0.0280 |
| Purine metabolism | 68 | 1.4066 | 2 | 0.4152 | 1 | 1 | 0.0129 |
| Steroid hormone biosynthesis | 70 | 1.4479 | 1 | 0.7770 | 1 | 1 | 0.0067 |
